# Advances in the Genetics and Molecular Biology of Brain Arteriovenous Malformations

**DOI:** 10.1007/s12975-026-01436-7

**Published:** 2026-04-18

**Authors:** Takahiro Tsuchiya, Satoru Miyawaki, Hideaki Ono, Hiroki Hongo, Shotaro Ogawa, Yu Sakai, Yudai Hirano, Daisuke Sato, So Hirata, Satoshi Koizumi, Nobuhito Saito

**Affiliations:** Department of Neurosurgery, Faculty of Medicine, The University of Tokyo, 7-3-1 Hongo, Bunkyo-ku, Tokyo, 113-8655 Japan

**Keywords:** Brain arteriovenous malformation (bAVM), Endothelial-to‐mesenchymal transition (EndMT), Somatic mutations, RAS/MAPK pathway, Therapeutic targets

## Abstract

Brain arteriovenous malformations (bAVMs) are high-flow vascular lesions characterized by direct arteriovenous shunting without an intervening capillary bed. The identification of somatic *KRAS* and *BRAF* mutations in sporadic bAVM endothelial cells (ECs) has fundamentally reshaped current understanding of bAVM biology, indicating that activation of the RAS/MAPK pathway drives aberrant angiogenic programs. In parallel, advances in genomic technologies have led to recognition of bAVMs as dynamic lesions that undergo ongoing vascular remodeling. Comprehensive transcriptomic profiling, including single-cell RNA sequencing, has uncovered distinctive molecular signatures in bAVM ECs, including heightened angiogenic and inflammatory signaling, endothelial-to-mesenchymal transition–like features, and loss of normal arteriovenous identity. Furthermore, animal models with EC-specific expression of mutant *KRAS* or *BRAF* exhibit bAVM-like lesions, which support the hypothesis that hyperactivation of the RAS/MAPK pathway is a key driver of lesion formation. These insights have accelerated the development of mechanism-based therapeutic strategies, and MEK and BRAF inhibitors targeting the RAS/MAPK pathway have shown promising results in preclinical studies. However, clinical translation remains challenging because of low variant allele frequencies and limited access to lesional tissue for genetic testing. Future approaches combining minimally invasive sampling methods, such as endovascular biopsy and peripheral blood cell-free DNA analysis, with ultra-sensitive detection technologies are expected to help overcome these limitations. Taken together, accumulating genetic evidence and a growing understanding of the inflammatory and immune microenvironment provide an important foundation not only for a deeper understanding of bAVM pathobiology but also for the development of future targeted therapies.

## Introduction

Brain arteriovenous malformations (bAVMs) are high-flow vascular lesions characterized by direct arteriovenous shunting without an intervening capillary bed [1]. Although relatively rare, bAVMs have an estimated prevalence of approximately 0.01% in the general population and represent a major cause of intracerebral hemorrhage in young adults, with substantial neurological morbidity and mortality [2]. The annual risk of hemorrhage is estimated to be 2% in unruptured bAVMs [3–5], increasing to 5% after an initial rupture [6]. Current management strategies including microsurgical resection, endovascular embolization, and stereotactic radiosurgery aim to achieve complete obliteration of the nidus but carry significant treatment-related risks depending on the location and angioarchitecture of the lesion [7, 8].

Historically, bAVMs have been regarded as congenital vascular anomalies arising from errors in cerebrovascular development and have long been conceptualized as structurally static lesions [9, 10]. This framework has guided both clinical decision-making and research efforts for decades. However, several clinical observations such as de novo lesion formation [11], post-treatment recurrence [12], and heterogeneous clinical behavior among morphologically similar lesions are difficult to fully reconcile with a purely congenital and static model [13].

Over the past decade, advances in genetic and molecular analyses have fundamentally reshaped our understanding of bAVM pathogenesis. The identification of somatic activating mutations in genes such as Kirsten rat sarcoma viral oncogene homolog (*KRAS)* and B-Raf proto-oncogene, serine/threonine kinase (*BRAF*), which are restricted to endothelial cells (ECs), provided compelling evidence that bAVMs are driven by genetic alterations and aberrant intracellular signaling [14–18]. In parallel, transcriptomic studies including bulk and single-cell RNA sequencing (scRNA-seq) have revealed profound alterations in EC state, inflammatory signaling, and vascular cell-cell interactions within bAVM lesions [19, 20].

These findings suggest that bAVMs cannot be fully understood as inert structural anomalies alone, but instead reflect complex biological processes involving genetic alterations, dysregulated signaling pathways, and inflammation. In this review, we summarize the advances in the genetics and molecular biology of bAVMs and discuss how these findings shape current concepts of bAVM pathophysiology, with an emphasis on endothelial dysfunction and progressive vascular remodeling.

## Pathophysiology

### Conceptual Shift in bAVM Pathophysiology

Traditionally, bAVMs have been regarded as congenital and static vascular anomalies [9, 10]. However, accumulating evidence suggests that de novo bAVMs can form and progress through repeated vascular remodeling, prompting a substantial revision of this long-standing concept [11–13, 21–26]. This shift is supported by evidence from clinical observations, histopathological and molecular findings, and experimental animal models.

Clinical observations, such as the occurrence of de novo bAVMs and of post-treatment recurrence or regrowth, suggest that bAVMs can develop after birth. New bAVMs have been documented years after normal cerebral angiography was confirmed [11, 21]. Additionally, recurrence or enlargement after apparent complete obliteration has been observed, suggesting that bAVMs are biologically active lesions rather than purely structural abnormalities [12]. Importantly, recurrence after apparent angiographic cure is not uniform across age groups. Recurrence appears to occur predominantly in pediatric patients, whereas it is uncommon in adults. In a cohort study of patients undergoing microsurgical resection with angiographic cure, recurrence was observed primarily in children, particularly among those who initially presented with hemorrhage [27]. Consistent with this observation, a systematic review and meta-analysis reported pooled recurrence rates of approximately 10% in pediatric brain AVMs [28]. In contrast, recurrence in adults appears substantially less frequent, although sporadic cases have been documented in the literature [29].

Histopathological and molecular findings also suggest that bAVM tissue exhibits EC activation and an inflammatory microenvironment, consistent with ongoing biological activity. ECs within bAVMs show enhanced proliferative and migratory capacity compared with normal brain vascular endothelium, along with dysregulation of angiogenesis-related signaling pathways [11, 22]. Moreover, the upregulation of multiple inflammatory mediators and the presence of chronic inflammatory microenvironment with immune cell infiltration have been demonstrated [23, 24]. These findings support the concept that bAVMs can be maintained and may progress through the reciprocal amplification of angiogenic and inflammatory programs.

Notably, inflammatory signatures and immune cell infiltration have been detected even in unruptured bAVM tissues, suggesting that inflammation may contribute to lesion activity rather than representing merely a secondary response to hemorrhage [23, 24]. These observations further support a model in which angiogenic cues, hemodynamic stress, and immune activation cooperate to promote de novo lesion formation and post-treatment regrowth.

In animal models, de novo AVM-like lesions can be induced in adult animals under defined conditions, thus providing an experimental model of acquired lesion formation. Chen et al. showed that combining EC-selective Activin A receptor like type 1 (*ALK1*) deletion with intracerebral Vascular endothelial growth factor (*VEGF*) stimulation in adult mice induces lesions resembling human bAVMs [25]. Similarly, Walker et al. showed that EC-selective *ALK1* deletion with VEGF overexpression produces bAVM-like vascular abnormalities [26].

Because experimental models that faithfully recapitulate prenatal human bAVM development remain limited, most current models induced in juvenile or adult animals are best interpreted as models of de novo lesion formation triggered by defined genetic perturbations combined with permissive angiogenic or inflammatory stimuli [25, 26].

### Vascular Remodeling and Fragility

Beyond ECs, mural cells, such as pericytes and vascular smooth muscle cells (VSMCs), contribute to vascular remodeling and lesion progression in bAVMs [30]. Reduced mural coverage is a key pathobiological axis that is linked to increased vascular permeability, vessel wall fragility, and hemorrhage risk.

Endothelial activation represents a central driver of these remodeling processes. In particular, pro-angiogenic signaling mediated by VEGF and its receptors has been implicated in abnormal endothelial proliferation and vascular destabilization in bAVMs [22]. Genetic and transcriptomic studies have identified perturbations in VEGF/VEGFR signaling pathways in sporadic bAVM lesions, supporting the concept that pro-angiogenic cues cooperate with underlying genetic alterations to promote aberrant angiogenesis and vascular remodeling [22]. Experimental studies further suggest that angiogenic stimulation, including VEGF signaling, can act as a permissive factor that facilitates lesion formation in genetically susceptible vascular beds [25].

In particular, pericytes are essential for stabilizing cerebral microvessels and maintaining barrier function [31]. bAVMs show reduced pericyte number and decreased vessel coverage, with more pronounced loss in lesions associated with microhemorrhage or rupture [31]. Consistently, lower mural coverage has been associated with increased vascular leakage and higher hemorrhage risk [30, 32]. In most experimental and histological studies, this phenomenon primarily reflects defective recruitment of pericytes. Such coverage defects may be exacerbated by the hemodynamic environment within the nidus, characterized by high-flow shunting and abnormal shear stress [31].

A leading hypothesis is that impaired mural cell recruitment during bAVM angiogenesis underlies this loss of coverage [30, 32]. During lesion formation, EC–pericyte crosstalk is required for basement membrane assembly and normal vessel caliber; inadequate pericyte support can promote luminal enlargement and dysmorphic vessel architecture [32]. In this context, Platelet-derived growth factor subunit B (PDGFB)/Platelet-derived growth factor receptor beta (PDGFRβ) signaling is a central regulator of mural recruitment, and reduced expression of *PDGFB* or *PDGFRβ* has been proposed to contribute to impaired mural cell mobilization and vascular instability in bAVMs [33, 34].

In addition to pericytes, VSMCs provide structural reinforcement and contractile regulation in larger vessels and arterioles. Histopathological studies of human bAVMs have reported abnormalities in smooth muscle investment and reduced expression of smooth muscle markers, suggesting impaired mural maturation and vessel wall integrity [30]. Loss or dysfunction of VSMCs may further weaken the vascular wall and contribute to hemorrhagic susceptibility [30]. Recent single-cell analyses of human cerebrovascular tissue also suggest that inflammatory programs within bAVM lesions may be associated with smooth muscle cell depletion and vascular wall instability [19].

The abnormal hemodynamic forces characteristic of bAVMs are integral to lesion formation and progression. Quantitative studies indicate that flow conditions can be altered by therapeutic interventions [35], and such mechanical stimuli may amplify angiogenesis and inflammation through endothelial mechanotransduction. Indeed, oscillatory shear stress has been reported to increase expression of *AGP2*, *AQP1*, and *TGFβR1* in AVM-derived ECs and to promote the transcription of angiogenesis-related genes [36].

## Genetic Mutations

The genetic architecture of bAVM can be divided into familial and sporadic. Although the vast majority of bAVMs (> 95%) are sporadic and occur without a family history [1, 3, 37], a subset arises in the context of inherited vascular anomaly syndromes such as hereditary hemorrhagic telangiectasia (HHT) and capillary malformation–arteriovenous malformation syndrome (CM-AVM) [38]. Representative familial entities include HHT, caused by germline mutations in *ENG* (*endoglin*) or A receptor like type 1 (*ACVRL1*, *ALK1*), and CM-AVM, associated with variants in RAS p21 protein activator 1 (*RASA1*) or EPH receptor B4 (*EPHB4*). In these inherited vascular anomaly syndromes, the Knudson’s “two-hit” model is increasingly supported [39], in which an underlying germline mutation is followed by a somatic second hit in local ECs, resulting in biallelic inactivation of the causal gene [40].

### HHT

HHT is an autosomal dominant vascular disorder primarily caused by pathogenic variants in *ENG*, *ACVRL1*, and SMAD family member 4 (*SMAD4*), corresponding to HHT1, HHT2, and juvenile polyposis (JP)-HHT, respectively [41–43]. More recently, pathogenic variants in *GDF2* (*BMP9*) have been reported in rare cases with an HHT-like vascular anomaly syndrome, suggesting an additional candidate gene; however, its overall contribution appears limited [44–46].

bAVMs occur significantly more often in HHT1 (13.4%) than in HHT2 (2.4%) [47]. *ENG* and *ACVRL1* do not show clear mutational hotspots in HHT [40]. Most HHT-associated variants are considered loss-of-function mutations. However, because these germline variants are present throughout the body, germline heterozygosity alone may not fully explain why telangiectasias and AVMs arise as focal lesions in specific vascular beds. Accordingly, a Knudson-type “two-hit” model has been proposed in HHT [39, 40], in which a local somatic event inactivates the remaining allele in affected tissue and thereby promotes lesion formation. Consistent with this model, low-frequency somatic variants and chromosomal loss of heterozygosity (LOH) have been detected as “second hit” in telangiectatic lesions from HHT patients, supporting a requirement for biallelic loss of *ENG*/*ACVRL1* in lesion formation [48, 49].

### CM-AVM

CM-AVM is an autosomal dominant vascular anomaly syndrome characterized by multiple small capillary malformations, with a subset of patients developing high-flow lesions such as AVMs and arteriovenous fistulas (AVFs) [50]. Germline loss-of-function variants in *RASA1* and *EPHB4* underlie CM-AVM1 and CM-AVM2, respectively [51, 52]. In CM-AVM1, most variants are truncating mutations in *RASA1*, and no mutational hotspot has been identified [51]. CM-AVM2 shares clinical features such as capillary malformations and high-flow vascular anomalies, but appears to have a lower overall association with high-flow lesions than CM-AVM1 [52]. The frequency of AVM/AVF has been reported as 31% in *RASA1* mutation carriers and 18% in *EPHB4* mutation carriers, with intracranial involvement in 10% and 3%, respectively [52].

As in HHT, the “second hit” model has been proposed for CM-AVM, in which germline *RASA1*/*EPHB4* variants provide the background and endothelial-level somatic events drive focal lesion development [51, 53, 54]. Additional evidence supporting somatic second hits affecting *RASA1* in ECs further implicates this mechanism as a basis for the localized nature of lesions [54].

### Sporadic bAVM

Since the late 2010s, the identification of lesion-restricted somatic mutations in a substantial proportion of sporadic bAVMs has fundamentally reshaped our understanding of disease biology [14]. In particular, the discovery that activating mutations in genes of the RAS/MAPK pathway are enriched in lesional ECs provides strong evidence that genetic alterations play a causal role in sporadic bAVM formation.

In 2018, Nikolaev et al. identified activating *KRAS* mutations (G12V, G12D, Q61H) in surgically resected bAVM lesions [14]. These variants were shown to localize predominantly to ECs within the nidus, positioning MAPK/Extracellular signal-regulated kinase (ERK) activation as a core pathogenic feature of sporadic bAVM. Subsequent replication studies suggest that *KRAS* mutations are detectable in approximately half of sporadic bAVMs [55].

In addition to *KRAS*, activating *BRAF* mutations have been identified. Hong et al. identified activating *BRAF* mutations (V600E) in spinal AVMs and bAVMs, along with two novel *KRAS* variants (G12A and S65 A66insDS) [15]. Priemer et al. reported a *KRAS* G12C mutation in bAVM [56]. In a cohort of 16 specimens, Goss et al. detected somatic mutations in 10 (63%), including *KRAS* (G12D/G12V) and *BRAF* (V600E/Q636X) [57]. A meta-analysis estimated the prevalence of somatic *KRAS* and *BRAF* mutations in bAVMs to be 55% and 7.5%, respectively [55]. By contrast, while additional candidate variants have been reported through whole-exome sequencing (WES) or broad targeted panels, no recurrent somatic mutations outside *KRAS*/*BRAF* have been consistently reproduced across independent cohorts [2, 15, 57]. In a study interrogating 295 genes within the RAS/MAPK pathway, variants were identified in genes other than *KRAS*/*BRAF*, but none were recurrent [2]. Previously reported somatic mutations in sporadic bAVM are summarized in Table 1.

**Table 1:** Summary of previous studies on genetic mutations in sporadic brain arteriovenous malformations

1^st^ Author, Year [Ref]	Sample size	Analysis methods (VAF)	KRAS G12V	KRAS G12D	KRAS G12A	KRAS G12C	KRAS Q61H	BRAF V600E	BRAF Q636X	Not detected
Nikolaev SI, 2018 [14]	72 bAVMs	WES (0.6–4.2%), ddPCR (0.3–6.7%)	43.1%	19.4%	0%	0%	1.4%	0%	0%	37.5%
Hong T, 2019 [15]	31 AVMs (21 brain and 10 spinal)	NGS panel (0.6–8.8%), ddPCR (0.03–7.3%)	45.2%	22.6%	3.2%	3.2%	3.2%	6.5%	0%	12.9%
Goss JA, 2019 [57]	16 bAVMs	MIP-seq (0.2–3.8%), ddPCR (0.3–2.8%)	31.3%	18.8%	0%	0%	0%	6.3%	6.3%	37.5%
Priemer DS, 2019 [56]	21 bAVMs	PCR (NA)	0%	23.8%	0%	4.8%	0%	0%	0%	71.4%
Oka M, 2019 [68]	38 bAVMs	ddPCR (0.6–12.3%)	39.5%	26.3%	0%	0%	0%	0%	0%	34.2%
Gao S, 2021 [2]	70 bAVMs	WES (NA), ddPCR (0.04–4.99%)	18.6%	37.1%	0%	0%	0%	0%	0%	44.3%

bAVM: brain arteriovenous malformation, BRAF: B-Raf proto-oncogene, serine/threonine kinase, ddPCR: droplet digital polymerase chain reaction, KRAS: kirsten rat sarcoma viral oncogene homolog, MIP-seq: molecular inversion probe sequencing, NA: not available, NGS: next-generation sequencing, PCR: polymerase chain reaction, VAF: variant allele frequency, WES: whole-exome sequencing

With respect to clinical correlations, Goss et al. and Gao et al. independently examined associations between somatic mutations and phenotypic features of sporadic bAVM, but did not identify significant relationships with age, sex, initial presentation, lesion location, or nidus size [2, 57]. Nevertheless, other studies have suggested potential links, including an association between *KRAS* mutations and hemorrhagic presentation [58], and an inverse relationship between variant allele frequency (VAF) and nidus size [15]. Thus, the clinical implications of these mutations remain incompletely defined.

Hemorrhage represents a clinically important phenotype in bAVM. However, genotype–phenotype correlations between somatic mutations and hemorrhagic presentation remain inconsistent across cohorts. For example, targeted sequencing studies have reported no clear differences in presenting symptoms, including hemorrhage, between mutation-positive and mutation-negative lesions or between KRAS- and BRAF-mutated cases [56, 57]. Mechanistic insights from experimental models nevertheless support a potential link between RAS/MAPK activation and hemorrhagic vulnerability. Endothelial KRAS activation in murine models induces bAVM-like lesions accompanied by neuroinflammation, and recent studies suggest that inflammatory myeloid cell accumulation may precede hemorrhagic conversion [59]. These observations suggest that hemorrhage in bAVM likely reflects complex interactions between genetic drivers, inflammatory programs, and vascular wall remodeling processes, including endothelial-to-mesenchymal transition (EndMT)-related destabilization discussed in later sections.

Both *KRAS* and *BRAF* are well-established components of the RAS/MAPK pathway, which regulates cellular proliferation, differentiation, and angiogenesis. Introduction of activating *KRAS* mutations into human ECs increases expression of angiogenesis-related genes and Notch pathway components, and enhances migratory capacity [58]. In zebrafish and murine models, endothelial-specific activation of *KRAS* induces AVM-like lesions [16]. Moreover, in a mouse model using a brain EC–tropic adeno-associated virus (AAV) vector, Mitogen-activated protein kinase kinase (MEK) inhibition suppressed progression of bAVMs [60]. For *BRAF*, somatic introduction of *BRAF* V600E into mouse brain endothelium has likewise been shown to recapitulate bAVM-like pathology [61].

Germline susceptibility factors for sporadic bAVM have also been explored. Candidate association studies have reported links between bAVM and single-nucleotide polymorphisms (SNPs) in genes such as *IL6*, *TNFα*, and *ACVRL1* [62]. However, a large genome-wide association study did not identify SNPs significantly associated with bAVM [63]. In contrast, a case–unaffected-parent trio WES analysis by Li et al. reported de novo germline variants in *ENG*, *JUP*, *EXPH5*, and *EPAS1*, raising the possibility that rare de novo variants may contribute to risk in at least a subset of cases [58].

### Extracranial (Peripheral) AVMs

While this review focuses on brain arteriovenous malformations (bAVMs), extracranial (peripheral) AVMs provide an informative comparator because both conditions converge on similar developmental and signaling pathways, yet differ in the dominant genetic drivers involved [64]. In extracranial AVMs, somatic variants most frequently affect the RAS/MAPK signaling pathway, with *MAP2K1* (*MEK1*) mutations representing the most commonly reported genetic alteration [65]. Additional mutations affecting other components of the same pathway, including *KRAS*, *HRAS*, and *BRAF*, have also been reported in extracranial vascular malformations [66, 67].

In contrast, sporadic bAVMs are predominantly driven by activating somatic mutations in *KRAS*, and less frequently in *BRAF*, that are restricted to endothelial cells [14, 15]. These findings indicate that although both extracranial (peripheral) AVMs and bAVMs converge on dysregulation of the RAS/MAPK pathway, the specific mutational nodes involved differ across vascular beds.

One possible explanation is that tissue-specific endothelial states, vascular bed–dependent hemodynamic environments, and local microenvironmental constraints, including the specialized neurovascular unit of the brain, influence which oncogenic signals most effectively drive abnormal angiogenesis and arteriovenous shunting. Differences in the dominant genetic drivers may also contribute to distinct therapeutic vulnerabilities and should therefore be considered when extrapolating therapeutic strategies across AVM locations [64].

## Challenges in Mutation Detection

Somatic mutations identified in sporadic bAVMs often occur at very low VAF, typically only a few percent and frequently below 1% within lesional tissue [2, 14, 15, 57, 68]. The mutation is enriched in a small subset of lesional ECs, whereas many surrounding mural and stromal cells remain genetically normal [14]. Moreover, mutant ECs may be distributed heterogeneously within the nidus, creating sampling bias; depending on the biopsy location and tissue volume, false-negative results can occur [2, 14]. At very low VAF, an additional challenge is distinguishing true somatic variants from sequencing-related artifacts [69].

Accurate detection of low-frequency variants therefore requires high-sensitivity approaches. To date, many studies have combined deep sequencing (whole-exome or targeted panels) with droplet digital PCR (ddPCR) for orthogonal validation and enhanced detection. Nikolaev et al. confirmed *KRAS* variants discovered by WES using ddPCR and further identified additional *KRAS* mutations in samples that were negative by WES due to low allele fractions [14]. Similarly, Hong et al. reported a high prevalence of *KRAS*/*BRAF* mutations in brain and spinal AVMs by integrating ultra-deep sequencing of a 422-gene panel with ddPCR [15].

A major practical limitation is that molecular profiling has largely depended on surgically obtained tissue. However, in a substantial proportion of patients, bAVMs are not amenable to resection or are managed without surgery, limiting access to lesional material. To reduce this dependence on resection material, an endoluminal biopsy approach has been described, in which cells are collected from within lesional vessels during cerebral angiography for downstream molecular profiling [70]. This strategy yielded in vivo transcriptomic signatures consistent with bAVM-associated pathways, including the RAS/MAPK pathway [70]. In addition, as a tissue-independent approach to molecular diagnosis, liquid biopsy using circulating cell-free DNA (cfDNA) has been explored in the AVM field [71], and a recent study further demonstrated that hotspot *KRAS* mutations (G12D/G12V) can be detected in plasma cfDNA from patients with bAVM using ddPCR [72]. This approach may be particularly important for the clinical implementation of molecular targeted therapies in bAVM, because tissue biopsy is generally infeasible and actionable mutations must be identified in living patients. In the future, combining such minimally invasive sampling with ultra-sensitive DNA assays may help move toward routine assessment of somatic mutations in living patients, which could facilitate patient stratification and molecular monitoring during targeted treatment.

## Molecular Signaling Pathways

### RAS/MAPK Pathway

**Fig. 1:**
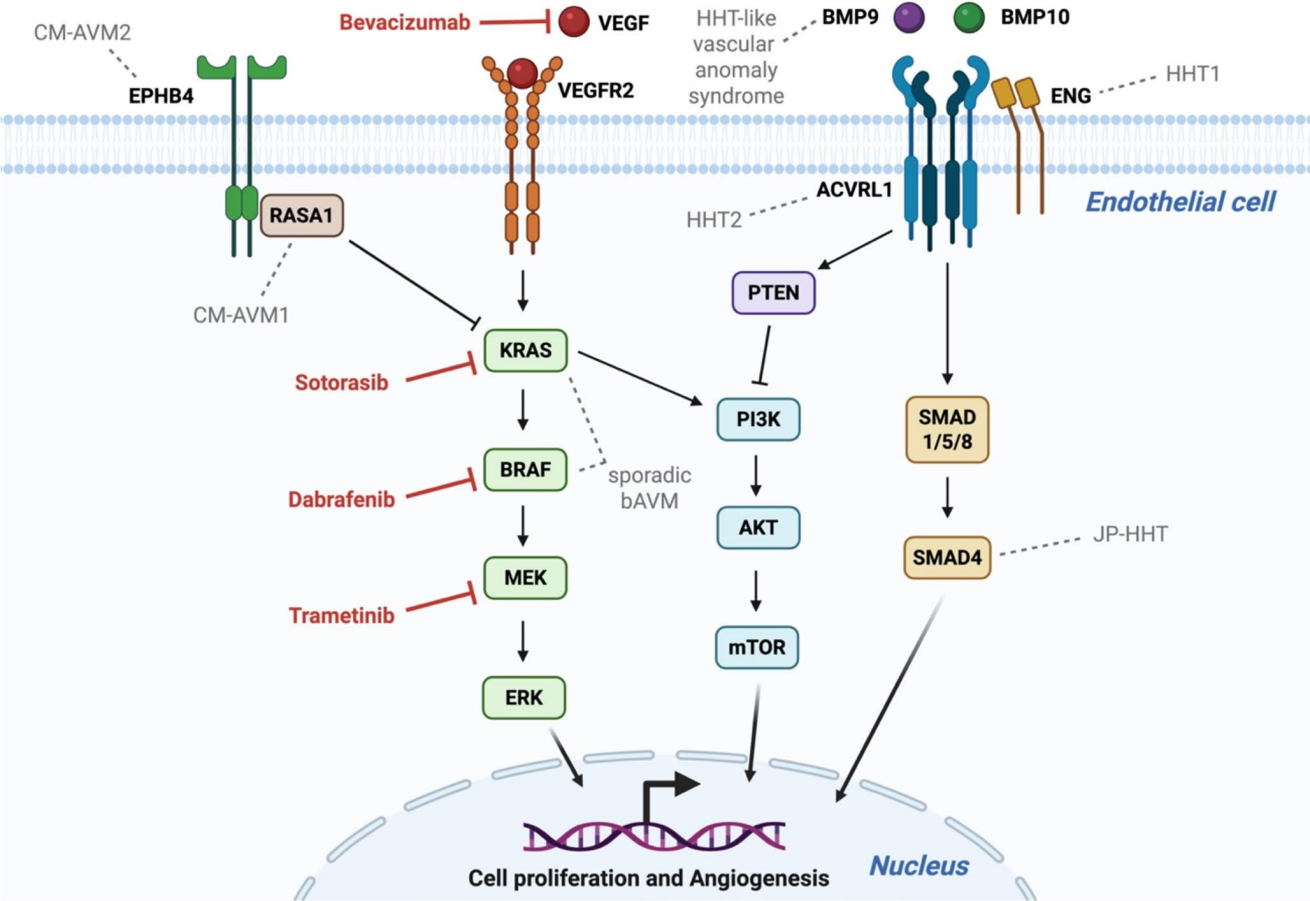
Schematic overview of endothelial signaling pathways involved in the pathogenesis of brain arteriovenous malformations (bAVMs). (i) Activation of the RAS/MAPK pathway induces MEK1/2–ERK1/2–mediated transcriptional programs that promote angiogenesis and cell proliferation. In sporadic bAVMs, this is driven primarily by gain-of-function mutations in *KRAS* or *BRAF*, whereas in familial capillary malformation–AVM (CM-AVM), loss-of-function mutations in *RASA1* or *EPHB4* are associated with dysregulated RAS/MAPK signaling and lesion formation. (ii) In the Transforming growth factor-β (TGF-β)/Bone morphogenetic protein (BMP) pathway, BMP9/BMP10 ligands signal through the ENG–ACVRL1 receptor complex, activating SMAD1/5/8–SMAD4 to regulate endothelial function and promote angiogenesis and cell proliferation. HHT-related genes are indicated: HHT1: *ENG*; HHT2: *ACVRL1*; JP-HHT: *SMAD4*; HHT-like vascular anomaly syndrome: *BMP9*. Representative therapeutic targets are indicated in red (bevacizumab, sotorasib, dabrafenib, and trametinib). Disease-associated genes are labeled in gray above the corresponding signaling components. Created with BioRender.com

The RAS/MAPK pathway is most prominently implicated in sporadic bAVM (Fig. 1). Across vascular malformations, high-flow lesions are more closely associated with activating mutations of the RAS/MAPK pathway [66], whereas slow-flow lesions (including venous, lymphatic, and cerebral cavernous malformations) more often involve activating mutations in the PI3K/AKT/mTOR pathway [73–75]. Gain-of-function mutations in *KRAS* or *BRAF* drive constitutive MEK1/2–ERK1/2 activation. Activated ERK1/2 translocates to the nucleus and stimulates a broad set of transcription factors, such as ELK1 and c-Fos, that regulate gene programs controlling proliferation, differentiation, and cell survival [76]. Persistent hyperactivation of the RAS–MAPK–ERK1/2 axis is a well-established mechanism in many cancers, including lung, colorectal, and pancreatic carcinomas [76]. In the context of bAVM, increased phospho-ERK1/2 has been observed in AVM-derived ECs, and introduction of mutant *KRAS* induces endothelial motility and angiogenesis-related transcriptional programs, supporting a functional role for MAPK overactivation in lesion formation [58].

Dysregulated RAS/MAPK pathway is also relevant to familial bAVMs. In CM-AVM1, loss-of-function variants in *RASA1* are thought to derepress *KRAS* activity, thereby causing pathological activation of the RAS/MAPK pathway [77]. Similarly, loss-of-function variants in *EPHB4* underlying CM-AVM2 have been linked to disease through disruption of RAS/MAPK pathway regulation [52].

Recent evidence suggests that oncogenic KRAS may also induce metabolic reprogramming in endothelial cells in bAVMs [78]. A recent study reported that KRAS-mutant endothelial cells exhibit increased glucose uptake and enhanced glycolytic flux, accompanied by increased membrane localization of glucose transporters such as GLUT1 and upregulation of hexokinase-2 (HK2) [78]. Inhibition of glycolysis suppressed pathological endothelial behaviors associated with sprouting angiogenesis and arteriovenous shunting, suggesting that metabolic remodeling may represent an additional functional layer downstream of the RAS/MAPK pathway.

### TGF-β/BMP Pathway

The TGF-β/BMP pathway is primarily associated with familial bAVM syndromes (Fig. 1). The major HHT genes (*ENG*, *ACVRL1*, and *SMAD4*) converge on endothelial signaling within the TGF-β/BMP superfamily. Endoglin (*ENG*) and ALK1 (*ACVRL1*) form a receptor complex on the EC surface that is activated by the ligands BMP9 (Growth differentiation factor 2, *GDF2*) and BMP10 [79, 80]. Downstream, this pathway signals through SMAD1/5/8 in partnership with SMAD4 to regulate transcriptional programs that maintain endothelial quiescence and vascular remodeling. In HHT, disruption of this signaling network is thought to impair normal angiogenic control and vessel wall maturation, thereby promoting vascular malformation formation [79, 81].

### Notch Signaling

Notch signaling represents another experimentally validated pathway involved in bAVM pathogenesis. Endothelial expression of constitutively active Notch4 is sufficient to induce AVM-like shunts in multiple vascular beds, and suppression of Notch4 signaling can lead to regression of these lesions, indicating a degree of reversibility [82]. Subsequent studies demonstrated that sustained Notch4 activation promotes enlargement of capillary-like vessels and the formation of arteriovenous shunts in the brain [83].

Consistent with these experimental findings, components of the Notch signaling pathway have also been detected in human AVM tissues. Notch receptor expression has been reported in endothelial cells within AVM nidus specimens, suggesting a potential role in vascular remodeling and arteriovenous specification [84]. In addition, altered expression of Notch pathway receptors has been associated with hemorrhagic presentation in bAVM patients [85].

Genetic perturbation of the canonical Notch transcriptional mediator Rbpj further revealed that balanced Notch signaling is required for normal arteriovenous organization, as endothelial Rbpj deletion during postnatal development leads to abnormal arteriovenous shunting [86]. More recent work showed that targeted endothelial deletion of Rbpj can normalize established Notch4-driven brain AVMs, highlighting the potential reversibility of this signaling axis [87]. In addition, Notch4-driven AVM formation has been linked to nitric oxide–dependent reduction in arteriolar tone, providing a mechanistic connection between endothelial signaling and hemodynamic regulation [88].

## Gene Expression and Transcriptomics

### Microarray Analysis and Bulk RNA Sequencing

Transcriptome-wide studies using human bAVM tissue have consistently reported gene expression changes related to inflammatory signaling, angiogenesis, cell migration, cytoskeletal dynamics, and extracellular matrix (ECM) remodeling compared with normal brain vasculature [24, 89–93]. These observations have accumulated over time from early microarray studies to more recent bulk RNA sequencing (RNA-seq) analyses (Table 2).

**Table 2:** Summary of previous studies on gene expression in sporadic brain arteriovenous malformations

1^st^ Author, Year [Ref]	Sample size	Analysis methods	Summary of results
Hashimoto T, 2004 [89]	6 bAVMs and 5 temporal lobes	Microarray	Angiogenesis- and cell-adhesion–related pathways, including increased *VEGFA* and integrin αvβ3 expression were upregulated.
Sasahara A, 2007 [90]	5 bAVMs (nidus and draining vein)	Microarray	Not many genes differed between the nidus and the draining vein; among the few, *EFNA1*, a gene related to embryogenesis and angiogenesis, was upregulated in the nidus.
Takagi, 2014 [116]	11 bAVMs, middle cerebral artery, and a cortical tissue sample	Microarray	bAVM nidus tissue showed a shared expression signature that could be categorized into death-related (e.g., *MMP9*, *LIF*, *SOD2*), neuron-related (e.g., *NPY*, *NeuroD2*, *CAMK2A*), and inflammation-related programs (e.g., *PTX3*, *IL6*, *IL8*, *CXCL10*).
Huo R, 2019 [91]	51 bAVMs (14 high-flow rate bAVM and 37 low-flow rate bAVM)	Bulk RNA-seq	Low-flow rate bAVMs showed activation of canonical Wnt signaling, with upregulation of *FZD10* and *MYOC*.
Hauer AJ, 2020 [24]	12 bAVMs and 16 intracranial arteries	Bulk RNA-seq	Inflammatory activation and upregulation of cytoskeletal/cell-migration programs, together with broad downregulation of genes involved in extracellular matrix/basement membrane composition, the angiopoietin–TIE system, and TGF-β signaling (including *SMAD4*).
Thomas JM, 2021 [93]	10 bAVMs and 10 vascular brain tissues	Bulk RNA-seq	Retinoic acid signaling activation with upregulation of *ALDH1A2* and the angiogenic target *CYR61/CCN1*.
Li H, 2022 [92]	65 bAVMs (28 ruptured and 37 un-ruptured)	Bulk RNA-seq	Ruptured bAVMs showed upregulation of genes related to inflammation and extracellular matrix organization/remodeling, with downregulation of cell-adhesion and myofibril assembly programs.
Winkler EA, 2022 [70]	4 bAVMs	Bulk RNA-seq (Endoluminal biopsy)	Endoluminal biopsy enabled endothelial cell–enriched RNA-seq profiling of unruptured bAVMs in living patients, identifying 106 DEGs enriched for the RAS/MAPK pathway. Biopsy profiles closely matched patient-matched surgical tissue, and integration with CFD linked wall shear stress and embolization-associated flow changes to inflammatory pathway upregulation.
Winkler EA, 2022 [19]	5 bAVMs and 5 cortical tissue samples /39 bAVMs (26 ruptured and 13 unruptured)	scRNA-seq/Bulk RNA-seq	Nidus endothelial cells show suppressed capillary/venule transcriptional identity with enrichment of arterial and venous programs, alongside pathologic endothelial transformations, abnormal vascular patterning, and vascular-derived inflammation with enhanced vascular–immune interactions.
Wälchli T, 2024 [20]	5 bAVMs/7 bAVMs and 3 temporal lobes	scRNA-seq/Bulk RNA-seq	Altered arteriovenous differentiation with reactivation of fetal programs and an immune-like endothelial shift marked by MHC class II upregulation.
Liu Y, 2025 [95]	1 bAVMs and 3 superficial temporal arteries	scRNA-seq	Increased monocytes and decreased smooth muscle cells, with dysregulated inflammation- and cell migration–related gene programs and altered cell–cell communication.

bAVM: brain arteriovenous malformation, CFD: computational fluid dynamics, DEG: differentially expressed gene, MHC: major histocompatibility complex, RNA-seq: RNA sequencing, scRNA-seq: single-cell RNA sequencing, TGF-β: transforming growth factor-β

Early microarray work showed increased *VEGFA* expression together with upregulation of integrin αvβ3, implicating angiogenic and adhesion-related programs in bAVM biology [89]. In a microarray comparison of the nidus and the draining vein, only a small number of transcripts differed; among them, *EFNA1*, a gene involved in embryogenesis and angiogenesis, was upregulated in the nidus [90].

Bulk RNA-seq analyses have further highlighted transcriptional signatures consistent with an inflammatory environment and reduced vascular wall integrity, suggesting that bAVMs may exist in a molecular environment that predisposes the vessel wall, which is already exposed to high-flow shunting, to destabilization [24]. A bulk RNA-seq analyses stratified by hemodynamic features reported activation of Wnt signaling in low-flow rate bAVMs, with increased expression of *FZD10* and *MYOC* [91]. In comparisons between ruptured and unruptured bAVMs, the ruptured group showed higher expression of gene sets related to inflammatory processes and ECM organization [92]. In addition, bulk RNA-seq analyses identified upregulation of the angiogenesis regulator *CYR61*/*CCN1*, supporting a coordinated transcriptional state linking inflammation, angiogenesis, and vascular wall remodeling [93].

### scRNA-seq

Evidence that bAVMs adopt endothelial programs distinct from normal brain vessels includes a study reporting expression of lymphatic-associated gene signatures, introducing the concept of unstable or reprogrammed vascular identity [94]. This finding suggests that bAVM endothelium is not merely hyperproliferative or hypermigratory, but may undergo broader transcriptional changes affecting lineage and functional identity. To interrogate these identity shifts at higher resolution, scRNA-seq approaches have enabled more granular interrogation of these changes (Table 2). In a scRNA-seq analysis of one bAVM and three controls, Liu et al. observed increased monocytes and reduced VSMCs, along with dysregulated expression programs and cell–cell communication pathways related to inflammation and cell migration [95]. Although limited by sample size, these observations are consistent with the concept that bAVM endothelium exists within an immune-interactive niche and that vascular–immune communication is perturbed. Winkler et al. compared adult control cerebrovascular cells (74,535 cells from five individuals) with cells from five unruptured bAVMs (106,853 cells) and found that nidus ECs exhibited reduced capillary/postcapillary venule identity with features of arterialization and venousization. They further reported pathological endothelial transformation, abnormal vascular patterning, and a vascular-derived inflammatory program coupled to immune cell interactions [19]. Wälchli et al. generated a large human brain vascular scRNA-seq atlas spanning fetal development through adulthood and including diseased vessels (117 samples; 606,380 cells). Within diseased vasculature, including bAVMs, they observed disrupted arteriovenous differentiation, reactivation of fetal gene expression programs, and immune-related changes such as increased endothelial MHC class II expression [20]. These scRNA-seq analyses advance the field from bulk-level signatures to cell-type–resolved mechanisms and support the view that bAVM vessels are characterized by profoundly altered cellular states and intercellular interactions.

### Endothelial-to-Mesenchymal Transition (EndMT)

One mechanistic framework to explain the distinctive endothelial state in bAVMs is EndMT. EndMT is a process in which ECs downregulate their typical markers and adopt mesenchymal-like properties. This can contribute to vascular wall destabilization and increased rupture risk through fibrosis, loss of intercellular junctions, and ECM remodeling. In human bAVM tissue, evidence of EndMT has been observed: for instance, excess collagen deposition and elevated PAI-1, along with increased expression of transcription factors like KLF4 and SNAI1/SNAI2, and upregulation of mesenchymal markers including VIM (vimentin), ACTA2 (α-smooth muscle actin), and S100A4 [94]. These changes support the presence of EndMT-associated remodeling in the bAVM vessel wall.

In unruptured bAVM, microhemorrhage has been proposed to portend a higher risk of future rupture and to represent a transitional state along the continuum of vascular destabilization [96]. In this study, EndMT-associated transcriptional programs were enriched in microhemorrhagic lesions, and SMAD6 downregulation was linked to activation of the TGF-β/BMP pathway, suggesting that EndMT may contribute directly to vascular wall fragility and the microhemorrhage phenotype.

Consistent with this concept, cell-resolution analyses have linked inflammatory myeloid programs to mural cell loss, including smooth muscle cell depletion that is associated with rupture and brain hemorrhage [19]. In KRAS-driven experimental models, microglia/macrophage accumulation correlates with blood–brain barrier leakage and precedes hemorrhagic conversion, and modulation of myeloid responses attenuates intracerebral hemorrhage incidence [59].

These findings collectively support a model in which EndMT-associated endothelial remodeling, inflammatory signaling, and mural cell destabilization interact to promote vascular fragility and hemorrhagic progression in bAVM.

Emerging data further suggest that EndMT may correlate with clinical vulnerability. Fu et al. reported that EndMT features positively correlate with microhemorrhage in bAVM, that *SMAD6* is reduced in microhemorrhagic lesions, and that EndMT in ECs can be promoted through activation of the TGF-β/BMP pathway [96]. These findings imply that EndMT may represent not only a transcriptional signature but also a disease axis linked to wall instability and the microhemorrhage phenotype.

**Fig. 2:**
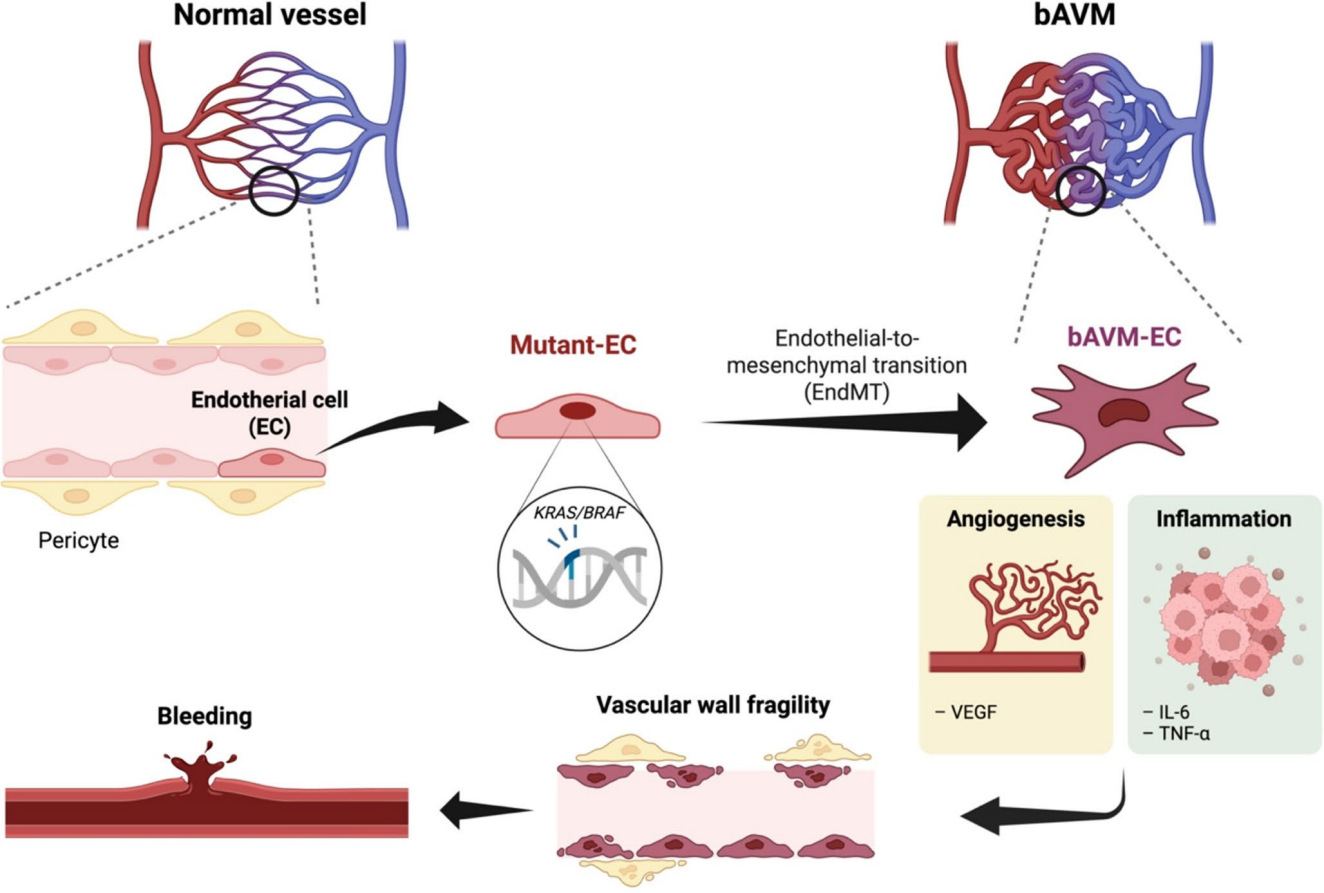
Conceptual model of mutant endothelial cells (ECs) and endothelial-to-mesenchymal transition (EndMT)-mediated vascular wall fragility in sporadic brain arteriovenous malformations (bAVMs). In normal vessels, ECs are supported by mural vascular components, which maintain vascular structure and integrity. In sporadic bAVMs, ECs harboring *KRAS*/*BRAF* mutations undergo EndMT and acquire a bAVM-like endothelial state. During this process, expression of endothelial markers is downregulated, whereas mesenchymal markers are upregulated. These changes are associated with pericyte loss, enhanced inflammation, and increased angiogenesis, which together drive vascular wall remodeling and destabilization. As a result, progressive vascular wall fragility increases the risk of hemorrhage. Created with BioRender.com

Mechanistically, activation of the RAS/MAPK pathway has been proposed as a trigger for EndMT and vascular wall remodeling. Sun et al. reported enrichment of Notch-related genes (*NOTCH1*, *HES1*, *HEY2*), induction of remodeling-associated genes (*VEGFA*, *VEGFC*, *DUSP5*, *HLX*), and upregulation of EndMT-associated genes (*SNAI1*, *SNAI2*, *ZEB1*) in ECs expressing *KRAS* G12V [32]. Li et al. likewise suggested that endothelial-restricted KRAS activation contributes to lesion formation and maintenance, and that EndMT may be broadly observed in bAVM [58]. Xu et al. demonstrated that endothelial *KRAS* G12D induces EndMT via MAPK/ERK activation and identified an ERK1/2–TGF-β/BMP–SMAD4 axis as a downstream pathway [97]. Although the VAF of *KRAS* mutations in bAVM endothelium is often low, EndMT features can be widespread across the lesion. To reconcile this discrepancy, it has been proposed that a small fraction of mutant ECs may disrupt endothelial–endothelial communication, allowing the pathological state to propagate to neighboring ECs [97]. A conceptual model of mutant ECs and EndMT-mediated vascular wall fragility in sporadic bAVMs is illustrated in Fig. 2.

## Epigenetics and Non-Coding RNAs

From the perspective of gene regulation, epigenetic modifications and non-coding RNAs have attracted attention as additional regulatory layers in bAVM pathobiology [98–104]. Although systematic studies of epigenetic alterations in bAVM such as DNA methylation and histone modifications remain limited, it is biologically plausible that ECs exposed to chronic inflammation and abnormal hemodynamic stress acquire sustained transcriptional changes through epigenetic reprogramming [101].

A methylome analysis of bAVM-derived ECs reported aberrant methylation not only in *KRAS*/*RBPJ* but also in genes implicated in cell adhesion and endothelial–VSMC crosstalk, including *EPHB1*, as well as methylation changes involving long non-coding RNA (lncRNA) loci [98]. These findings suggest that epigenetic mechanisms may contribute to stabilization of disease-associated transcriptional states [98].

Research on non-coding RNAs in bAVM has largely focused on microRNAs (miRNAs). Circulating miRNA signatures potentially linked to VEGF-related pathways have been reported in patients with bAVM [99]. Dysregulation of broader non-coding RNA networks has also been proposed to modulate core signaling pathways including the RAS/MAPK pathway and thereby contribute to AVM formation [100]. Additional studies have identified bAVM-associated miRNA profiles, including decreased miR-18a, miR-137, and miR-195* and increased miR-7-5p, miR-199a-5p, and miR-200b-3p [101]. miR-18a is reduced in nidus-derived ECs and has been shown to suppress multiple angiogenic pathways, suggesting that its downregulation may promote aberrant vascular growth [102]. Reduced miR-137 and miR-195* expression has also been linked to pathological angiogenesis involving vascular wall cells [103]. More recently, miR-3131 carried in exosomes derived from ECs harboring *KRAS* G12D was shown to target *PICK1* and promote EndMT in neighboring *KRAS* wild-type ECs [104].

## Immune Microenvironment and Inflammation

bAVMs are characterized by a chronically inflamed microenvironment, and this inflammatory state is considered a key modifier of vascular wall fragility and hemorrhage risk. Immunohistochemical analyses of resected specimens have demonstrated inflammatory cell infiltration within the vessel wall and the perivascular brain parenchyma, supporting the presence of localized inflammation in bAVM lesions [105]. Consistent with these observations, scRNA-seq analyses have further suggested pathological transformation of nidus endothelium accompanied by coordinated interactions with immune cells [19]. In *KRAS* G12V–EC bAVM mouse model, three-dimensional immunostaining of cleared human and mouse bAVM tissues demonstrated an abundance of microglia/macrophages surrounding the bAVM nidus, and their presence was correlated with blood–brain barrier (BBB) leakage in the bAVM region. Moreover, long-term minocycline treatment to suppress microglia/macrophages activity reduced the incidence of hemorrhage [59]. Other *KRAS* G12V-EC models suggest that *KRAS*-mutant ECs can activate microglia/macrophages, and that inflammatory mediators released from these immune cells further amplify endothelial junctional disruption and BBB breakdown [106]. Across these studies, minocycline treatment has been associated with reduced inflammatory cytokine levels, restoration of VE-cadherin, and attenuation of BBB leakage, supporting the notion that inflammation control may contribute to vascular stabilization.

This inflammatory environment is accompanied by elevated inflammatory cytokines and adhesion molecules. Among these, Interleukin 6 (IL-6) and Tumor necrosis factor α (TNF-α) have been repeatedly linked to bAVM biology [107]. Increased IL-6 levels have been observed in lesional tissue of bAVM and may be higher in cases presenting with hemorrhage [108]. In addition, inter-individual variability in inflammatory responses may influence disease susceptibility and clinical behavior; in this context, polymorphisms in the *IL-6* gene have been suggested to contribute to bAVM risk and bleeding propensity [62]. A major molecular mechanism connecting inflammation to vascular destabilization involves degradation of the ECM and basement membrane by proteases such as matrix metalloproteinases (MMPs). In bAVM lesions, increased proteolytic activity including upregulation of MMP-9 has been reported [105, 108].

Beyond destabilizing the vessel wall, inflammation may promote angiogenesis and thereby contribute to lesion maintenance and growth. Inflammatory conditions are known to facilitate induction of pro-angiogenic factors, including VEGF, which is consistent with bAVM transcriptomic profiles [24]. This raises the possibility of a positive feedback loop in which inflammation and angiogenesis mutually reinforce one another. Experimental models support this framework: AVM-like lesions can be induced in the adult mouse brain by combining local *Alk1* deletion with VEGF-driven angiogenic stimulation [26], and similarly by applying an angiogenic stimulus in the setting of EC-selective *Alk1* deletion [25].

## Animal Models

Robust and reproducible animal models are essential to test the causal role of *KRAS*/*BRAF* mutations in bAVM pathogenesis and evaluate the therapeutic potential of molecularly targeted agents in vivo.

Fish et al. first established *KRAS*-driven bAVM models in mouse and zebrafish by introducing activating *KRAS* mutations selectively into ECs. Using an endothelial Cre driver, they induced *KRAS* G12D expression in brain ECs not only during the postnatal period but also in adult mice [16]. Approximately 50% of animals in both age groups developed bAVMs within 8 weeks, indicating that AVM formation can occur even after the cerebrovascular network has matured and is therefore not restricted to developmental angiogenesis. In transgenic zebrafish embryos expressing *KRAS* G12V, arteriovenous shunts formed in ~ 50% of animals. These shunts were reversible with MEK inhibition but resistant to PI3K inhibition, supporting the RAS/MAPK pathway as a primary driver.

Multiple studies have since evaluated the MEK inhibitor trametinib in *KRAS*-driven bAVM mouse models [60, 109, 110]. Park et al. developed a sporadic bAVM model using a brain EC–tropic AAV to deliver *KRAS* G12V (AAV-BR1-*KRAS* G12V) [60]. Retro-orbital venous sinus injection of AAV-BR1-*KRAS* G12V reproducibly induced bAVMs in all animals within 9 weeks. The resulting lesions recapitulated key morphological features of human bAVMs, including dilated vessels and a tangled nidus-like architecture composed of feeding arteries and draining veins. In this model, trametinib suppressed lesion development when administered at early stages of bAVM formation. Nguyen et al. reported that pan-endothelial induction of *KRAS* G12D produced early-onset vascular abnormalities, and that trametinib treatment after lesion establishment improved survival and attenuated vascular pathology [109]. Myint et al. further showed that trametinib reduced hemorrhage burden and EndMT, accompanied by improved survival in *KRAS* G12V-induced bAVM mice [110]. 

Tu et al. developed the first mouse model of sporadic bAVM driven by somatic *BRAF* V600E in brain ECs using AAV-BR1-Cre [61]. A major advantage of this platform is that AAV dose and injection site can be precisely controlled, enabling generation of diverse bAVM phenotypes with respect to lesion size, location, and hemorrhagic severity. Pharmacologically, *BRAF* inhibition significantly suppressed *BRAF* V600E-driven bAVM formation when administered during early stages, whereas therapeutic efficacy was diminished in established lesions. These data suggest that while RAS/MAPK pathway activation is critical for initiating abnormal vascular morphogenesis, subsequent growth after arteriovenous shunt formation may not be strictly dependent on continued pathway activation.

Fraissenon et al. used a *KRAS* G12C model and reported that treatment with the *KRAS* G12C inhibitor sotorasib reduced bAVM formation and mortality [111]. 

## Molecular Targeted Therapies

As discussed above, studies using animal models have identified multiple candidate molecular therapies for bAVM. In particular, the somatic activating mutations in *KRAS* and *BRAF* found in sporadic bAVMs are well-established oncogenic drivers in cancer, and drugs targeting these pathways have already been approved in oncology. Accordingly, the most promising strategy for mechanism-based therapy in bAVM is inhibition of the RAS/MAPK pathway driven by *KRAS*/*BRAF* mutations.

The MEK inhibitor trametinib has shown encouraging effects in preclinical models [60, 109, 110]. In humans, however, evidence remains limited and is currently largely restricted to case reports and ongoing clinical trials. For extracranial AVMs, off-label trametinib use in a *KRAS*-mutant case was associated with a therapeutic response, including reduced blood flow [112]. In addition, a phase II trial for complex extracranial AVMs (NCT04258046) and a phase II pilot study of trametinib for surgically treated unruptured AVMs of the brain and body (NCT06098872) are currently underway.

For *BRAF* mutations, the BRAF inhibitor dabrafenib may represent a potential therapeutic option [113]. In extracranial AVMs, a case with *BRAF* V600E was treated off-label with dabrafenib in combination with trametinib, resulting in marked lesion regression after 3 months [114]. In contrast, data on efficacy in bAVM remain scarce and require further investigation. Murine studies suggest that while early administration can suppress disease progression, therapeutic benefit may be reduced once lesions are established, indicating that timing of intervention could be a critical issue for clinical translation [61]. 

The *KRAS* G12C-specific inhibitor sotorasib is Food and Drug Administration (FDA)-approved for the treatment of advanced non–small cell lung cancer harboring *KRAS* G12C. Targeting *KRAS* G12C is therefore an attractive strategy for AVMs with this genotype. Two adults with severe extracranial AVMs carrying *KRAS* G12C reportedly experienced rapid symptomatic improvement and lesion shrinkage after sotorasib treatment [111]. However, because *KRAS* G12C appears to be rare in bAVM, the proportion of eligible patients is likely limited.

Beyond RAS/MAPK pathway inhibition, anti-angiogenic strategies have also been explored. A first-in-human phase I trial evaluated the safety and efficacy of the anti-VEGF antibody bevacizumab in sporadic bAVM, but did not observe a significant reduction in bAVM volume at 52 weeks (NCT02314377) [115]. A phase II/III trial is planned to assess bevacizumab in symptomatic bAVM patients (NCT06264531).

## Conclusion and Future Directions

In this review, we synthesize recent advances in bAVM genetics and molecular biology, highlighting their implications for pathophysiology, endothelial dysfunction, and vascular remodeling. The accumulating evidence supports the concept that bAVM is a dynamic and multifactorial lesion that can emerge and evolve through the interplay of somatic mutations, chronic inflammation, and hemodynamic stress. In particular, the discovery of somatic mutations within lesional ECs represents a major breakthrough in elucidating mechanisms of lesion formation and endothelial dysfunction. Moreover, growing data implicate dysregulated signaling pathways, aberrant transcriptional programs, and immune-mediated processes, further deepening our understanding of bAVM pathobiology and its vascular remodeling programs. Although the full disease mechanism remains incompletely defined, integrating these complementary lines of evidence may enable a more coherent view of bAVM as a unified biological entity. At present, no molecular targeted therapy has been established for bAVMs; however, recent advances suggest that bAVMs may become actionable targets guided by molecular information. Continued efforts are needed to strengthen translational research and to build a robust evidence base that supports the development and evaluation of new therapeutic strategies.

## Data Availability

No datasets were generated or analyzed during the current study.

## Abbreviations

ACVRL1: activin A receptor like type 1
ALK1: activin A receptor like type 1
AAV: adeno-associated virus
AVF: arteriovenous fistula
bAVM: brain arteriovenous malformation
BBB: blood–brain barrier
BMP: bone morphogenetic protein
BRAF: B-Raf proto-oncogene, serine/threonine kinase
cfDNA: circulating cell-free DNA
CM-AVM: capillary malformation–arteriovenous malformation syndrome
ddPCR: droplet digital polymerase chain reaction
EC: endothelial cell
ECM: extracellular matrix
ENG: endoglin
EndMT: endothelial-to-mesenchymal transition
EPHB4: EPH receptor B4
ERK: extracellular signal-regulated kinase
FDA: food and drug administration
GDF2: growth differentiation factor 2
HHT: hereditary hemorrhagic telangiectasia
IL-6: interleukin 6
KRAS: kirsten rat sarcoma viral oncogene homolog
lncRNA: long non-coding RNA
LOH: loss of heterozygosity
MEK: mitogen-activated protein kinase kinase
miRNA: microRNA
MMP: matrix metalloproteinase
PDGFB: platelet-derived growth factor subunit B
PDGFRβ: platelet-derived growth factor receptor beta
RASA1: RAS p21 protein activator 1
RNA-seq: RNA sequencing
scRNA-seq: single-cell RNA sequencing
SMAD4: SMAD family member 4
SNP: single-nucleotide polymorphism
TGF-β: transforming growth factor-β
TNFα: tumor necrosis factorα
VAF: variant allele frequency
VEGF: vascular endothelial growth factor
VSMC: vascular smooth muscle cell
WES: whole-exome sequencing
